# Intraoperative haemoadsorption for antithrombotic drug removal during cardiac surgery: initial report of the international safe and timely antithrombotic removal (STAR) registry

**DOI:** 10.1007/s11239-024-02996-x

**Published:** 2024-05-06

**Authors:** Michael Schmoeckel, Matthias Thielmann, Kambiz Hassan, Stephan Geidel, Jan Schmitto, Anna L. Meyer, Keti Vitanova, Andreas Liebold, Nandor Marczin, Martin H. Bernardi, Rene Tandler, Sandra Lindstedt, Marijana Matejic-Spasic, Daniel Wendt, Efthymios N. Deliargyris, Robert F. Storey

**Affiliations:** 1 Department of Cardiac Surgery, Klinikum Grosshadern, Ludwig-Maximilians-University, Marchioninistr. 15, Munich, D-81377 Germany; 2 Department of Thoracic- and Cardiovascular Surgery, West German Heart and Vascular Center, Essen, Germany; 3 Department of Cardiac Surgery, Asklepios Klinik St. Georg, Hamburg, Germany; 4 Department of Cardiac-, Thoracic-, Transplantation- and Vascular Surgery, Hannover Medical School, Hannover, Germany; 5 Department of Cardiothoracic Surgery, University Hospital Heidelberg, Heidelberg, Germany; 6 Department of Cardiovascular Surgery, German Heart Centre, Munich, Germany; 7 Department of Cardiothoracic and Vascular Surgery, Ulm University Medical Center, Ulm, Germany; 8 Department of Anaesthesia, Royal Brompton Hospital, Royal Brompton & Harefield Hospitals, Part of Guy’s and St Thomas’ NHS Foundation Trust, London, UK; 9 Division of Cardiothoracic and Vascular Anaesthesia and Intensive Care Medicine, Medical University of Vienna, Vienna, Austria; 10 Department of Cardiac Surgery, University Hospital Erlangen, Erlangen, Germany; 11 Department of Cardiothoracic Surgery and Transplantation, Skåne University Hospital, Lund, Sweden; 12 CytoSorbents Europe GmbH, Berlin, Germany; 13 CytoSorbents Inc, Princeton, NJ USA; 14 Division of Clinical Medicine, University of Sheffield, Sheffield, UK; 15 NIHR Sheffield Biomedical Research Centre, Sheffield Teaching Hospitals NHS Foundation Trust, Sheffield, UK

**Keywords:** Haemoadsorption, Antithrombotic removal, Cardiac surgery, CytoSorb, Ticagrelor, DOAC

## Abstract

Intraoperative antithrombotic drug removal by haemoadsorption is a novel strategy to reduce perioperative bleeding in patients on antithrombotic drugs undergoing cardiac surgery. The international STAR registry reports real-world clinical outcomes associated with this application. All patients underwent cardiac surgery before completing the recommended washout period. The haemoadsorption device was incorporated into the cardiopulmonary bypass (CPB) circuit. Patients on P2Y_12_ inhibitors comprised group 1, and patients on direct-acting oral anticoagulants (DOAC) group 2. Outcome measurements included bleeding events according to standardised definitions and 24-hour chest-tube-drainage (CTD). 165 patients were included from 8 institutions in Austria, Germany, Sweden, and the UK. Group 1 included 114 patients (62.9 ± 11.6years, 81% male) operated at a mean time of 33.2 h from the last P2Y_12_ inhibitor dose with a mean CPB duration of 117.1 ± 62.0 min. Group 2 included 51 patients (68.4 ± 9.4years, 53% male), operated at a mean time of 44.6 h after the last DOAC dose, with a CPB duration of 128.6 ± 48.4 min. In Group 1, 15 patients experienced a BARC-4 bleeding event (13%), including 3 reoperations (2.6%). The mean 24-hour CTD was 651 ± 407mL. In Group 2, 8 patients experienced a BARC-4 bleeding event (16%) including 4 reoperations (7.8%). The mean CTD was 675 ± 363mL. This initial report of the ongoing STAR registry shows that the intraoperative use of a haemoadsorption device is simple and safe, and may potentially mitigate the expected high bleeding risk of patients on antithrombotic drugs undergoing cardiac surgery before completion of the recommended washout period.

Clinical registration number: ClinicalTrials.gov identifier: NCT05077124.

**Graphical Abstract:**

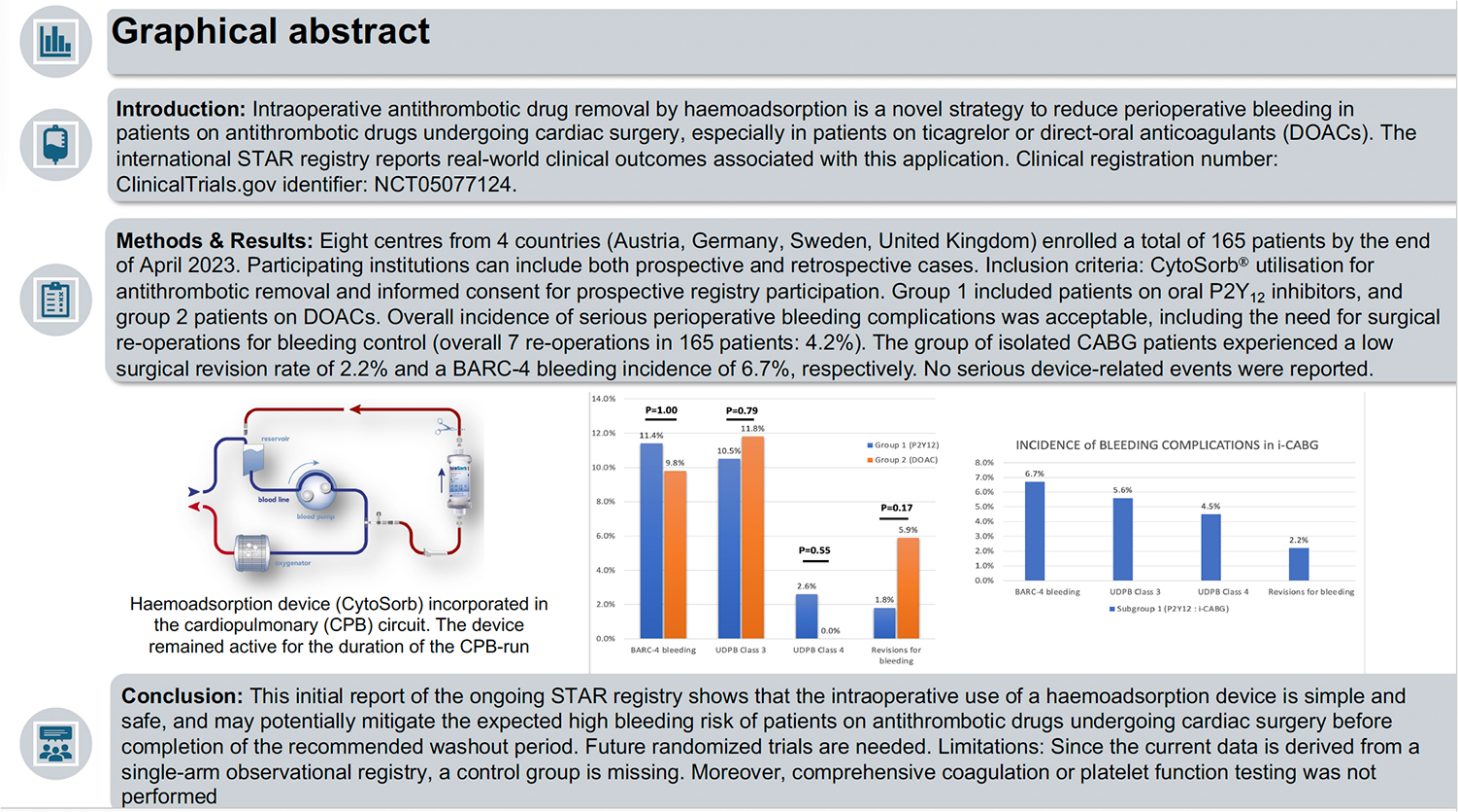

**Supplementary Information:**

The online version contains supplementary material available at 10.1007/s11239-024-02996-x.

## Introduction

Antithrombotic drugs are cornerstone therapies for patients with cardiovascular disease. Millions of patients receive chronic treatment with direct-acting oral anticoagulants (DOACs) to reduce stroke risk associated with atrial fibrillation or reduce recurrent events after venous thromboembolism [1]. In addition, P2Y_12_ inhibitors are routinely used in patients with acute coronary syndromes and after percutaneous coronary interventions [2–4]. The major safety risk associated with antithrombotic drugs is bleeding which can be either spontaneous or iatrogenic when such patients require urgent or emergent interventions including cardiac surgery [5].

Many of these antithrombotic drugs (e.g. DOACs and the reversibly binding P2Y_12_ receptor antagonist ticagrelor) can be effectively removed from the circulation with the use of a polymer bead haemoadsorption device and this novel approach is increasingly used to reduce perioperative bleeding in patients on a broad range of antithrombotic drugs undergoing urgent or emergency cardiac surgery [6–9]. The international Safe and Timely Antithrombotic Removal (STAR) registry is designed to collect real-world clinical outcomes associated with this application.

## Methods

The international Safe and Timely Antithrombotic Removal (STAR) registry is designed to collect high-fidelity data on patients who underwent intraoperative antithrombotic drug removal during cardiac surgery as part of their routine care. Participating institutions can include both prospective and retrospective cases. Collected clinical and resource utilisation data are entered in an electronic case report form (CRF). Safety is assessed by collection of definite, probable, or possible device-related adverse events. Data collection is done up to 30 days post-operation. The sponsor and funding source of the registry is CytoSorbents Inc., Princeton, NJ, USA.

### Ethical statement

This registry complies with the Declaration of Helsinki. National central or local approvals of respective Ethics committees were granted for the STAR registry according to local regulations (complete list available in the Supplement). Written consent was obtained before or after surgery from prospective patients and was waived for retrospective patients.

#### Inclusion criteria

CytoSorb^®^ utilisation for antithrombotic removal and informed consent for prospective registry participation.

#### Exclusion criteria

Use of CytoSorb® for purposes other than antithrombotic removal.

#### Patient groups

Group 1 included patients on oral P2Y_12_ inhibitors, and group 2 patients on DOACs.

#### Hemoadsorption therapy

**Fig. 1:**
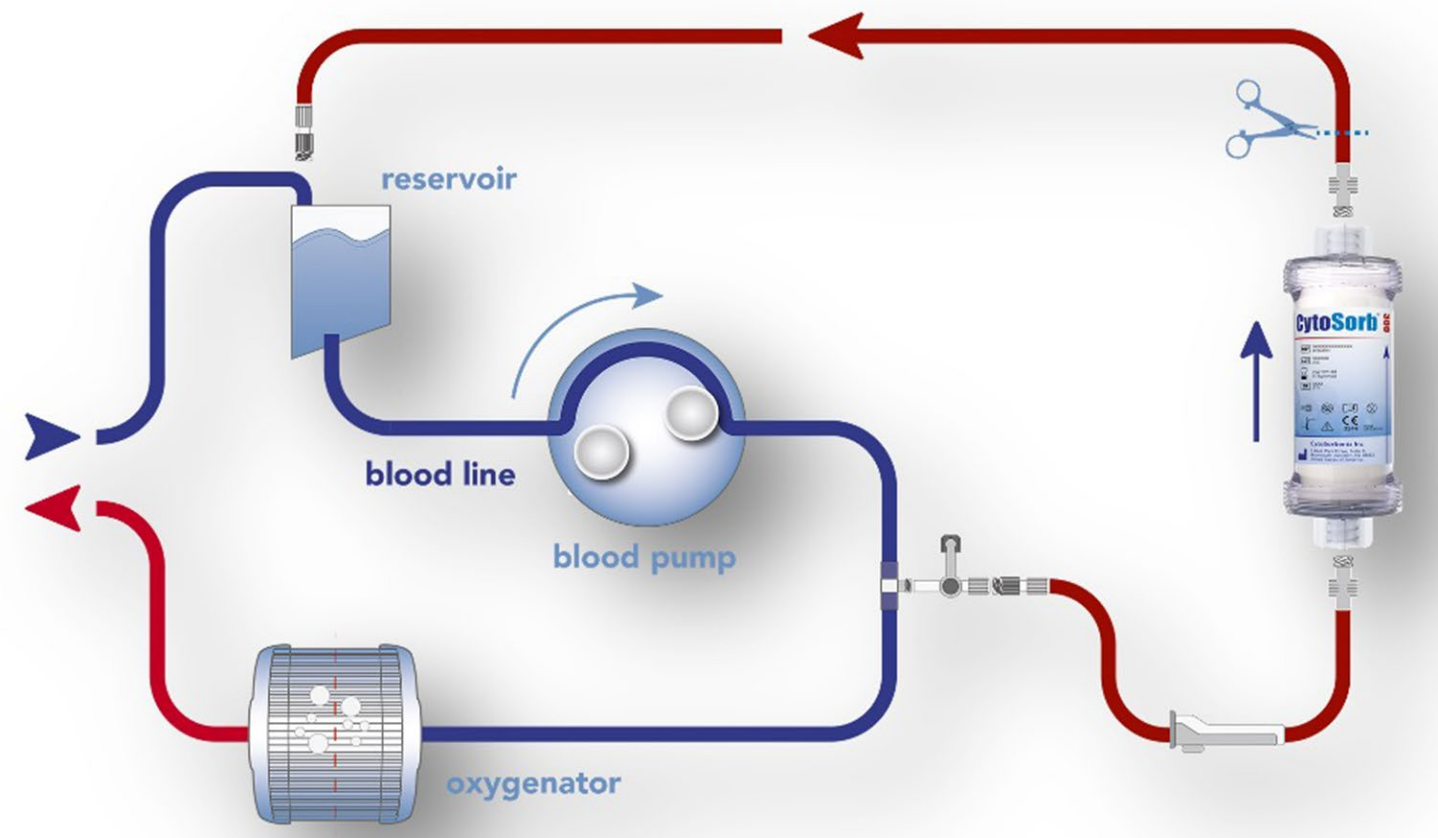
Haemoadsorption device (CytoSorb^®^) incorporated in the cardiopulmonary (CPB) circuit. The device remained active for the duration of the CPB-run

Antithrombotic removal via haemoadsorption therapy was performed with the CytoSorb® adsorber (CytoSorbents Inc., Princeton, NJ, USA). This CE-marked device is based on extracorporeal blood purification and is approved to remove ticagrelor and rivaroxaban. The cartridge is filled with highly biocompatible, porous polymer beads covered with a divinylbenzene coating and can be easily integrated into various extracorporeal circuits, such as e.g., continuous renal replacement therapy, extracorporeal membrane oxygenation (ECMO), or cardio-pulmonary bypass (CPB), as shown in Fig. 1. Each polymer bead is between 300 μm and 800 μm in size and has multiple pores and channels, giving it a large (> 40,000 m^2^) effective surface area for binding hydrophobic small and medium-sized molecules up to 60 kDa of molecular weight [10].

#### Outcome measures

Bleeding complications were recorded according to the *B*leeding *A*cademic *R*esearch *C*onsortium (BARC) and *U*niversal *D*efinition of *P*erioperative *B*leeding (UDPB) definitions. Additional outcomes included 24-hour chest tube drainage (CTD), detailed analysis of blood product transfusions, re-operation for bleeding, and in-hospital mortality. Safety of the device was assessed by investigator-reported adverse device events, including severity and related classifications. A detailed classification list is given in the Supplement.

## Results

Eight centres from 4 countries (Austria, Germany, Sweden, United Kingdom) enrolled a total of 165 patients by the end of April 2023.

**Fig. 2:**
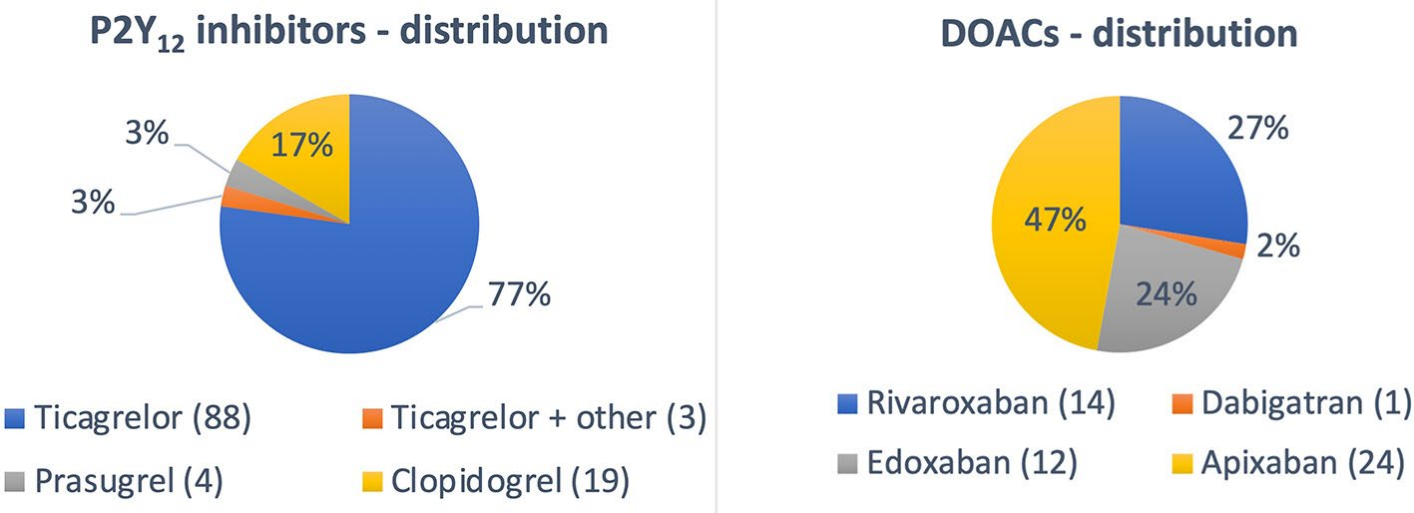
Drug distribution in group 1 (P2Y_12_ inhibitors) and group 2 (DOAC)

Significant baseline differences were noted between groups and 1 and 2 including age, gender, and qualifying diagnosis (Table 1). Patients on all 3 available P2Y_12_ inhibitors were included; however, the vast majority were treated with ticagrelor (Fig. 2). In group 2, almost half of the patients were on apixaban, and approximately one-quarter each were on either rivaroxaban or edoxaban (Fig. 2). All antithrombotic drugs were given according to their approved indication with a high prevalence of atrial fibrillation in group 2 (Table 1). The mean washout period in both groups was less than 48 h.

**Table 1:** Demographics

Variable	Group 1 (P2Y_12_ inhibitors) n = 114	Group 2 (DOAC) n = 51	*p*-value
Age, years	62.9 ± 11.6	68.4 ± 9.4	0.004
Gender, male	92 (80.7)	27 (52.9)	< 0.001
BMI, kg/m^2^	28.7 ± 5.6	30.5 ± 5.8	0.09
Acetylsalicylic acid	92 (80.7)	18 (35.3)	< 0.001
Acute coronary syndrome	92 (80.7)	12 (23.5)	< 0.001
Atrial fibrillation	17 (15.9)*	21 (48.8)^+^	< 0.001
Urgent indication (24-48h)	27 (23.6)	7 (13.7)	0.21
Emergency indication (< 24h)	41 (36.0)	6 (11.8)	0.001
NYHA functional class III/IV	27 (23.7)	17 (33.3)	0.25
Hypertension	90 (78.9)	38 (74.5)	0.54
Diabetes	39 (34.2)	18 (35.3)	1.00
Hyperlipidaemia	59 (51.8)	24 (47.1)	0.61
Smoking	38 (33.3)	8 (15.7)	0.02
Renal dysfunction (creatinine > 1.3mg/dL / failure (dialysis))	17 (14.9)	9 (15.7)	0.65
EuroSCORE-II, %	7.6 ± 11.2	8.3 ± 10.5	0.75

Data are presented as number (%) or mean±SD. * Incidence of atrial fibrillation was available in 107 patients from group 1 and for 43 patients from group 2^+^

BMI - body mass index, NYHA - New York Heart Association, EuroSCORE - European System for Cardiac Operative Risk Evaluation

**Fig. 3:**
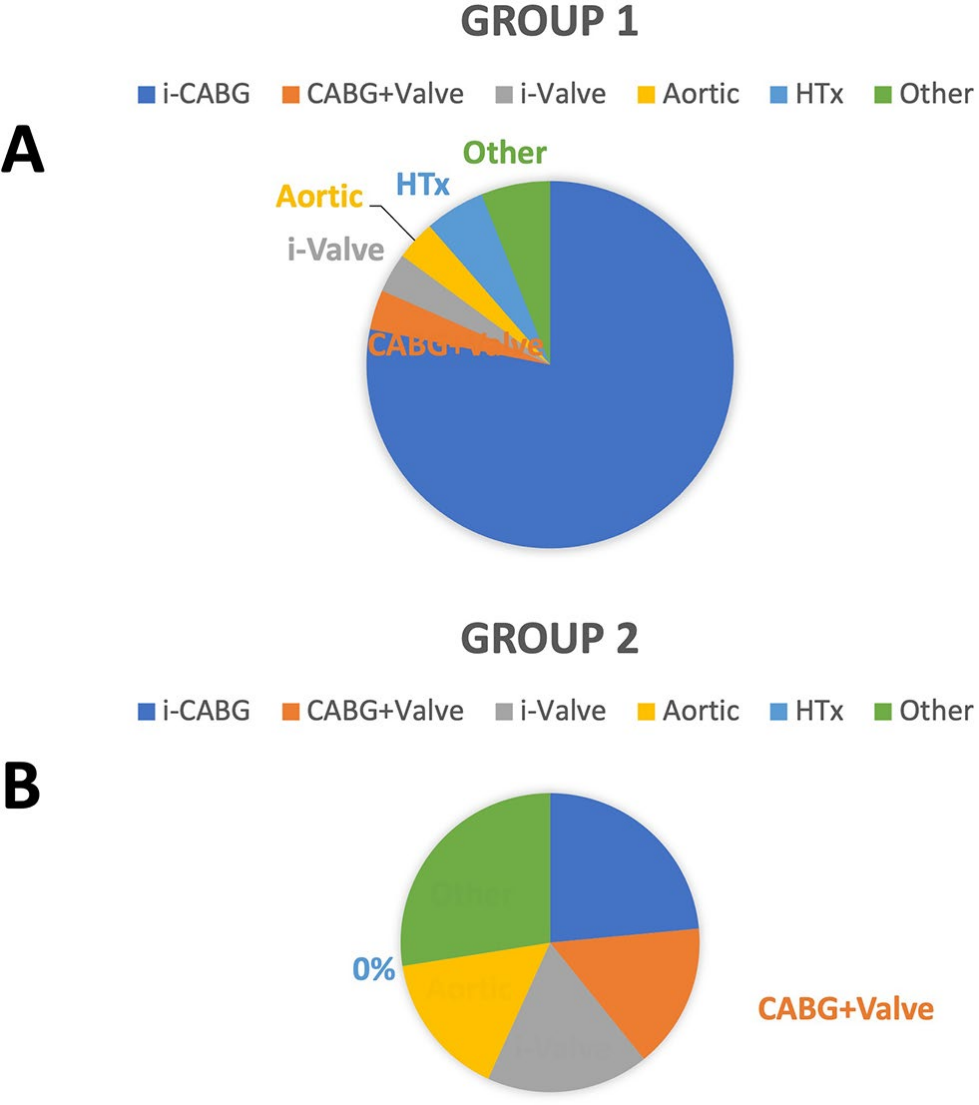
(**A**) Indications for cardiac surgery in patients on P2Y_12_ inhibitors (group 1) and (**B**) Indications for cardiac surgery in patients on DOACs *(i-CABG: isolated CABG, i-Valve: isolated heart valve surgery)*

Procedural outcomes are summarised in Table 2 and depicted in Fig. 3A and B. In Group 1, the vast majority of patients underwent isolated coronary artery bypass grafting (i-CABG) (Fig. 3A). In group 2, there was an almost equal distribution between i-CABG, combined procedures (CABG + heart valves), isolated heart valve procedures (i-Valve), and aortic surgery (Fig. 3B). The mean period between the last dose and surgery was shorter in group 1 compared with group 2 (33.2 ± 26.1 vs. 44.6 ± 33.1 h respectively, *p* = 0.05). Both CPB duration, which also represents device exposure time, and aortic cross clamp times were comparable and not significantly different between the two groups (Table 2). Moreover, in the i-CABG population no significant difference in regard to device exposure time was observed.

**Table 2:** Procedural outcomes

Procedural outcomes	Group 1 (P2Y_12_ inhibitors) *n* = 114	Group 2 (DOAC) *n* = 51	*p*-value
CPB time (device exposure), min.	117.1 ± 62.0	128.6 ± 48.4	0.28
ACC time, min.	72.5 ± 39.6	84.0 ± 41.5	0.15
i-CABG	89 (78.1)	12 (23.5)	< 0.001
i-CABG, CPB-time (device exposure)	98 ± 35	117 ± 49	0.13
CABG + valve(s)	4 (3.5)	8 (15.7)	0.008
i-Valve	4 (3.5)	9 (17.6)	0.003
Aortic surgery	4 (3.5)	8 (15.7)	0.008
Aortic surgery (Type A aortic dissection)	3 (2.6)	2 (3.9)	0.17
HTx	6 (5.3)	0 (0)	0.17
ECMO support	8 (7.0)	2 (3.9)	0.72
Impella® support	2 (1.8)	—	—
IABP support	3 (2.6)	—	—
TandemHeart® support	1 (0.9)	—	—
Washout period, h	33.2 ± 26.1	44.6 ± 33.1	0.05
Revision for bleeding	3 (2.6)	4 (7.8)	0.20
24-hour CTD, mL	651 ± 407	675 ± 363	0.75

Data are presented as number (%) or mean±SD

CPB - cardiopulmonary bypass, ACC - aortic cross clamp, i-CABG - isolated CABG, i-Valve - isolated heart valve surgery, HTx - heart transplantation, ECMO – extracorporeal membrane oxygenation, IABP – intra-aortic balloon pump, CTD - chest tube drainage

### Bleeding complications

**Fig. 4:**
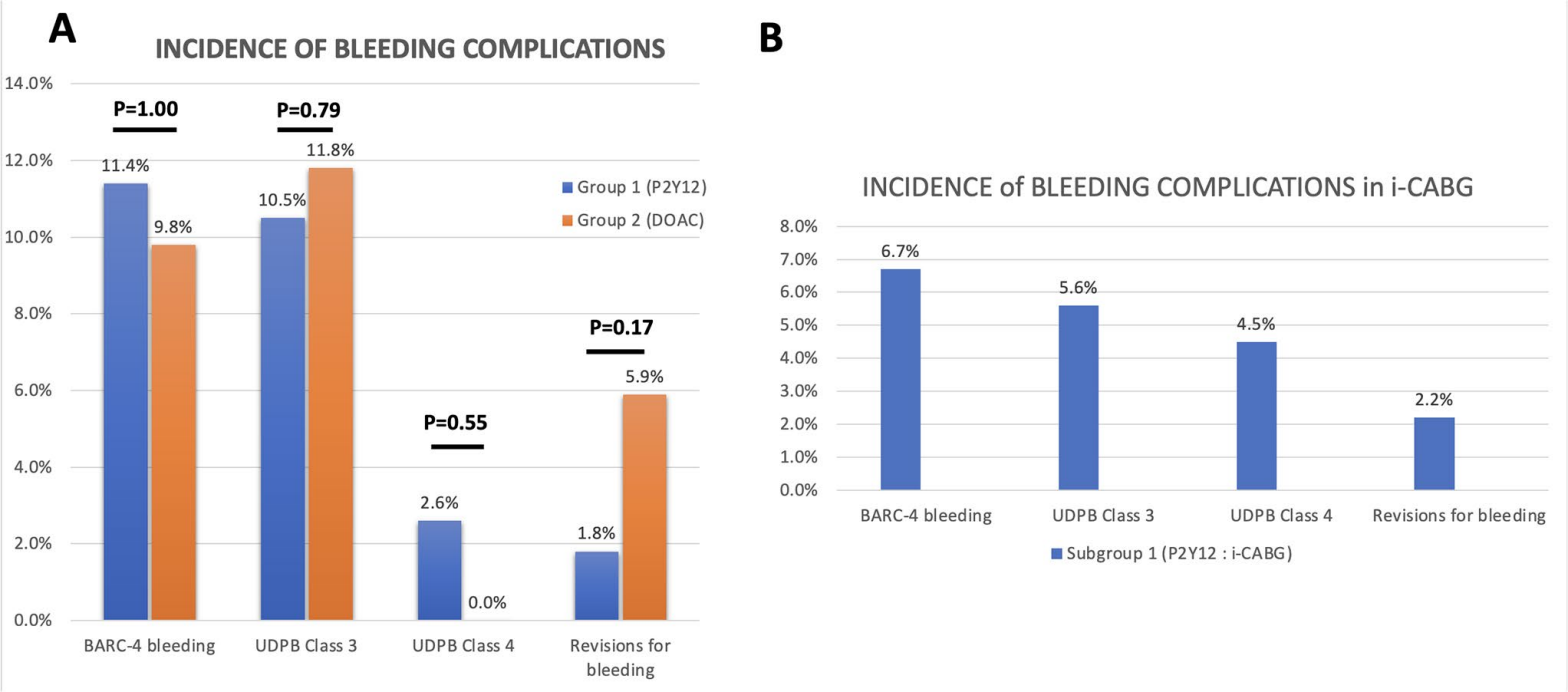
(**A**) Bleeding complications according to BARC-4 and UDPB and (**B**) Incidence of bleeding complications in i-CABG. *(BARC: Bleeding Academic Research Consortium; UDPB: Universal Definition of Perioperative Bleeding)*

Serious postoperative bleeding complications according to either BARC or UDPB definitions were not significantly different between the two groups (Fig. 4A). BARC-4 bleeding occurred in 13.2% in group 1 vs. 15.7% in group 2. Total 24-hour CTD also did not differ significantly between groups (Table 2). Blood product transfusions are summarised in Table 3. Packed red blood cells (pRBC) were not needed during the first 24-hours after surgery in 66% of group 1 and in 57% of group 2, while more than 5 units were required in only 2 patients in group 1. 75% and 84% patients in group 1, and 96% and 76% in group 2 did not require platelets or fresh frozen plasma (FFP), respectively. More than 5 units of platelets or FFP were needed in 3% of patients (6% of group 1 patients and no patients in group 2). Surgical revisions within 48 h due to ongoing bleeding were numerically higher in group 2 (7.8% vs. 2.6%; *p* = 0.2). Finally, patients in group 1 undergoing i-CABG represented the largest uniform subgroup in terms of type of antithrombotic drug and type of surgery. The rates of bleeding complications for this specific cohort are depicted in Fig. 4B. One severe postoperative bleeding event was caused by a bleeding left mammary artery side branch.

**Table 3:** Blood product consumption (postoperative day 1)

Variable	Group 1 (P2Y_12_ inhibitors) *n* = 114	Group 2 (DOAC) *n* = 51
No pRBC transfusion, n (%)	75 (66)	29 (57)
1–2 pRBC units, n (%)	27* (24)	20 (39)
3–4 pRBC units, n (%)	9* (8)	2 (4)
≥5 pRBC units, n (%)	2* (2)	0
No platelet transfusion, n (%)	85 (75)	49 (96)
1–2 platelet units, n (%)	21 (18)	2 (4)
3–4 platelet units, n (%)	5 (4)	0
≥5 platelet units, n (%)	3 (3)	0
No FFP transfusion, n (%)	96 (84)	39 (76)
1–2 FFP units, n (%)	6 (5)	10 (20)
3–4 FFP units, n (%)	5 (4)	2 (4)
≥5 FFP units, n (%)	7 (6)	0

Data are presented as number (%); *Quantity missing for one subject. pRBC - packed red blood cells, FFP - fresh frozen plasma

A total of 19 patients (17%) in the P2Y_12_ inhibitor group were treated by clopidogrel as the antithrombotic agent. These patients compared to the other 95 patients in Group 1 showed significant higher CTD (905 ± 433mL vs. 596 ± 386mL, *p* = 0.012), received numerically higher rates of platelet transfusions (36.8% vs. 23.2%, *p* = 0.25), and also experienced more UDPB class 3 bleeding events (*p* = 0.01). Of note, none of the included patients received recombinant factor VIIa (rFVIIa, NovoSeven^®^).

### Mortality

Mortality at 30 days in the overall registry cohort was 9.1% (15/165) with 7.0% in group 1 compared to 13.7% in group 2, showing no significant difference (*p* = 0.24). The corresponding preoperative EuroSCORE-II for the whole cohort was 7.6 ± 11.2% in group 1 and 8.3 ± 10.5% in group 2 (*p* = 0.75).

Non-survivors had very high preoperative risk with a EuroSCORE-II of 27 ± 19% compared to 6.0 ± 7.8% in survivors (*p* < 0.001), underwent more emergent/urgent procedures and required significantly more postoperative mechanical circulatory support with extracorporeal membrane oxygenation (ECMO) or TandemHeart^®^ compared to survivors (26.7% vs. 6.7%, *p* = 0.02). In the three patients receiving postoperative mechanical circulatory support, 2 BARC-4 bleeding events occurred, however without any surgical re-exploration. In the i-CABG cohort of group 1, the 4 deaths that occurred were all emergency procedures. A detailed breakdown of the mortalities is presented in Table 4. All deaths were cardiac-related and associated with low output states and multi-organ failure. No fatal bleeding leading to death occurred.

**Table 4:** Mortality-related data

Variable	Overall registry deaths, *n* = 15	Deaths in group 1, *n* = 8	Deaths in group 2, *n* = 7
30-day mortality	15 (9.1)	8 (7.0)	7 (13.7)
< 3 days	6 (40.0)	3 (37.5)	3 (42.9)
4–8 days	3 (20.0)	2 (25.0)	1 (14.3)
8–30 days	6 (40.0)	3 (37.5)	3 (42.9)
Urgency status			
Emergency	10 (66.7)	8 (100.0)	2 (28.6)
Urgent	4 (26.7)	0	4 (57.1)
Unknown	1 (6.6)	0	1 (14.3)
EuroSCORE-II, %	27 ± 19	32 ± 24	18 ± 15
Procedure			
i-CABG	7 (46.7)	4 (50.0)	3 (42.9)
CABG + valve(s)	2 (13.3)	2 (25.0)	0
Aortic dissection	1 (6.7)	1 (12.5)	0
Other	5 (33.3)	1 (12.5)	4 (57.1)
24-hours CTD, mL	760 ± 286	690 ± 240	783 ± 317
Mechanical circulatory support			
ECMO	3 (20.0)	2 (25.0)	1 (14.3)
TandemHeart®	1 (6.7)	1 (12.5)	0

Data are presented as number (%) or mean ± SD

EuroSCORE - European System for Cardiac Operative Risk Evaluation, (i)CABG – (isolated) CABG, CTD – Chest tube drainage, ECMO – extracorporeal membrane oxygenation

### Safety

All participating sites assessed the device as safe according to the ISO 14155:2020 classification (detailed classification given in the Supplement) and did not report any (serious) device related adverse events.

## Discussion

This initial report from the ongoing international STAR registry summarises the outcomes and bleeding complications in patients treated with oral antithrombotics undergoing cardiac surgery before the recommended washout period. The following main observations can be derived from the ongoing registry. First, the P2Y_12_ inhibitor group consisted mainly of ticagrelor patients, whereas in the DOAC group, apixaban was the most frequent drug prescribed. Second, the index operations differed between the groups due to the different underlying conditions requiring treatment with antiplatelets versus anticoagulants. Third, the overall incidence of serious perioperative bleeding complications was acceptable, including the need for surgical re-operations for bleeding control (overall 7 re-operations in 165 patients: 4.2%). Fourth, the overall 30-day mortality of 9% was high and likely related to the very high operative risk of emergent/urgent operations. Importantly, the removal of antithrombotics with intraoperative haemoadsorption was simple and safe, without any device-related adverse events reported.

In the current analysis, we sought to present data on two different groups: First, the group treated by preoperative P2Y_12_ inhibitors (consisting mainly of CABG patients) and a second group with preoperative DOAC treatment (consisting mainly of complex or combined surgeries). Both antithrombotic groups differed regarding their surgical indication and demographics. Group 2 included mainly elderly, high-risk patients undergoing more complex, long-lasting surgeries (including combination surgeries), or major aortic surgery (including aortic type A dissections). These patients are per se presenting with a high baseline risk for surgery and the pre-existing high risk for postoperative bleeding is further aggravated by the presence of antithrombotic agents.

Of note, 19 patients on clopidogrel have been included into the P2Y_12_ inhibitor group. It has to be acknowledged that since clopidogrel, in contrast to ticagrelor, is irreversibly bound to platelets, it remains unclear whether removal via haemoadsorption is to be expected. The serious bleeding rates reported in this analysis compare favourably to historical benchmarks. Patients on dual anti-platelet therapy (DAPT) with ticagrelor and acetylsalicylic acid undergoing isolated CABG surgery within 24 h after drug discontinuation have been reported with an incidence of BARC-4 bleeding of 38% and a 24-hour CTD of 813 ± 554mL, resulting in a re-exploration rate of 6.1% [11]. In a more recent study, a postoperative 24-hour CTD of 698 ± 409mL and a surgical re-exploration rate of 8.3% was reported [12] after a washout period of 24 h. In the current analysis, patients in group 1 undergoing isolated CABG had an incidence of BARC-4 bleeding of 4.5%, mean 24-hour CTD of 651 ± 407mL and a re-exploration rate of only 2.6%, therefore suggesting that intraoperative haemoadsorption may lower the high baseline bleeding risk of patients on P2Y_12_ inhibitors undergoing cardiac surgery before completing the recommended washout period. It should be acknowledged, however, that in the current analysis the washout period of the isolated CABG group was 33.2 h compared to 24 h in the dataset of Hansson et al. [11].

The European Multicenter Registry on Coronary Artery Bypass Grafting (E-CABG) reported a higher incidence of UDPB severe or massive bleeding in ticagrelor-treated patients when ticagrelor was discontinued 0–2 days, which corresponds to the current analysis with 1.4 days of washout in group 1. Holm et al. reported a UDPB class 3/4 bleeding rate of 16.0%, which was lower in the current analysis (UDPB class > 3: 10.1%). In addition, the current analysis also showed a lower incidence of BARC-4 bleeding events (6.7%) in the isolated CABG group compared to the E-CABG registry (11.8%) [5].

In a previous analysis of cardiac surgery patients under DOAC medications, it was demonstrated that an increased incidence of bleeding is observed up to 10 days after drug cessation [6]. Specifically in patients undergoing urgent aortic surgery, DOAC use was independently associated with increased perioperative mortality [13]. Current guidelines recommend that, when possible, patients should discontinue DOACs at least 2 days prior to surgery; however, surgeons routinely report that their standard washout period is longer [14].

Although surgical re-operation for bleeding control was required in only 7 out of 165 patients (4.2%) in the overall cohort, it appeared to be slightly more frequent in DOAC patients who underwent more complex and combined procedures. In a previous study, it was demonstrated that in patients on apixaban discontinued < 48 h before surgery, the 24-hour CTD was 893 ± 579mL with a re-thoracotomy rate of 8.3% [8]. In group 2 of the current STAR registry analysis, we observed a lower 24-hour CTD of 675 ± 363mL but noted a similar surgical re-exploration for bleeding rate of 7.8%. Since 24-hr CTD is a strong independent predictor of surgical outcomes including mortality [15, 16], the current results again support the use of intraoperative haemoadsorption in patients on DOAC undergoing cardiac surgery before completing the recommended washout period.

An overall mortality at 30 days of 9% in the overall cohort must be viewed in the context of a high percentage of urgent/emergent indications and also the complexity of the index surgical procedures. In addition, a fairly high number of patients in the current analysis required mechanical extracorporeal circulatory support in the perioperative period which historically defines patients with high perioperative mortality ranging between 15 and 25% [17, 18]. The preoperative assessed EuroSCORE-II in both groups tended to reflect, per its definition, a high-risk cohort (EuroSCORE-II 7.6 ± 11.2% in group 1 and 8.3 ± 10.5% in group 2). Specifically, among non-survivors the baseline EuroSCORE-II was 27 ± 19% and much higher compared to a value of 6.0 ± 7.8% in survivors, supporting the notion that mortality was directly linked to very high baseline risk. A recent published analysis analysed the bleeding events in patients undergoing surgical revascularisation in patients receiving dual-antiplatelet therapy < 72 h before surgery. They reported major bleeding events according to BARC-4 of 44.3% (10.5% surgical revisions) resulting in an in-hospital mortality of 9.0% [19].

It has been previously shown in a benchtop model that the CytoSorb^®^ haemoadsorption device (CytoSorbents, Inc., Princeton, NJ, USA) effectively reduces DOAC (apixaban and rivaroxaban) and ticagrelor levels in a time-dependent fashion [20]. It is therefore postulated that actively reducing DOAC or ticagrelor levels can lower the bleeding risk in such patients operated on before complete drug washout. These observations were recently validated by the results of a clinical study measuring ticagrelor levels before and after CPB during which haemoadsorption was utilised for antithrombotic removal [21]. In this first-in-human analysis, it was demonstrated that a mean CPB + haemoadsorption time of 97 ± 43 min led to a significant reduction in ticagrelor levels by 67% (*p* < 0.001). An additional important benefit of reduced circulating ticagrelor levels is that it may allow platelet transfusions to work more effectively. Previous reports have shown that platelet reactivity remained unchanged following transfusion of platelets to ticagrelor-treated patients [22], an observation likely explained by the reversible mode of binding of ticagrelor to platelets that renders newly transfused platelets also vulnerable to inhibition.

Whether intraoperative ticagrelor removal with haemoadsorption reduces perioperative bleeding in patients on ticagrelor undergoing cardiac surgery is currently investigated in the pivotal, double blind, randomised Safe and Timely Antithrombotic Removal – Ticagrelor (STAR-T) trial in the US and Canada (ClinicalTrials.gov Identifier: NCT04976530) [23].

In a case report, Dalmastri et al. described the successful preoperative reduction of apixaban levels in a patient scheduled for emergency bilateral nephrostomy by 48% after 150 min. of haemoadsorption during renal replacement therapy [24]. Therefore, analogous results are to be expected in further clinical trials aiming at significant DOAC removal before major surgery.

Importantly, no serious adverse device-related events were observed as classified by all investigators. This is in line with previous observations using the haemoadsorption device in different clinical settings [25]. Hence, the most recent ESAIC Guidelines for the management of severe perioperative bleeding [26] provided a class 2 C recommendation for the use of haemoadsorption as an adjuvant in patients on ticagrelor or rivaroxaban undergoing emergency cardiac/aortic surgery on cardiopulmonary bypass to reduce bleeding complications.

## Limitations

Our study has three major limitations that have to be considered when interpreting the results. First, since the current data is derived from a single-arm observational registry, a control group (without adsorber use) is missing. Future trials should analyse the current findings in a randomized or propensity-score matched fashion. Moreover, future trials should consider including coagulation or platelet function testing to determine the potential return of haemostatic activity after surgery. Second, comprehensive coagulation or platelet function testing results were not available to accurately determine the impact of the residual oral antithrombotics following cardiac surgery. Third, due to the “open” and all-comers real-world inclusion intention of the registry, results might be biased by the inclusion of many patients that would routinely be excluded from studies with strict inclusion and exclusion criteria (i.e. emergencies, high risk cases). Finally, we only presented the mandatory and complete data available for packed red blood cells or platelet transfusions without having detailed data on other blood products given (e.g. tranexamic acid, fibrinogen etc.).

## Conclusion

This initial report of the ongoing STAR registry shows that the intraoperative use of a haemoadsorption device may potentially mitigate the expected high bleeding risk of patients on antithrombotic drugs undergoing cardiac surgery before completion of the recommended washout period. Moreover, in patients on antithrombotic drugs undergoing cardiac surgery before the recommended washout period, the intraoperative use of hemoadsorption was reported by investigators to be easy to implement and generally safe. Whether active antithrombotic removal can reduce serious perioperative bleeding in patients undergoing urgent cardiac surgery compared to control subjects who are not treated with the device is currently being evaluated in the double-blind, randomized Safe and Timely Antithrombotic Removal-Ticagrelor (STAR-T) trial.

### Electronic supplementary material

Below is the link to the electronic supplementary material.

Supplementary Material 1

## Data Availability

The data underlying this article were provided by CytoSorbents Inc., Princeton, NJ, USA. Data will be shared on request to the corresponding author with permission of CytoSorbents.

## Abbreviations

ACC: Aortic Cross Clamp
BARC: Bleeding Academic Research Consortium
BMI: Body Mass Index
CABG: Coronary Artery Bypass Grafting
CPB: Cardio-pulmonary bypass
CRF: Case Report Form
CTD: Chest tube drainage
DOAC: Direct Oral Anticoagulant drug
ESAIC: European Society of Anaesthesiology and Intensive Care
HTx: Heart Transplantation
i-CABG: Isolated CABG
i-Valve: Isolated heart valve procedure
STAR: Safe and Timely Antithrombotic Removal
UDPB: Universal Definition of Perioperative Bleeding
