# Supplementary material for: Intraoperative haemoadsorption for antithrombotic drug removal during cardiac surgery: initial report of the international safe and timely antithrombotic removal (STAR) registry
Source: J Thromb Thrombolysis. 2024 May 6;57(6):888–97. doi: 10.1007/s11239-024-02996-x (PMC11315775; doi:10.1007/s11239-024-02996-x)
Supplement: Supplementary file 1 — Supplementary Material 1 [file 11239_2024_2996_MOESM1_ESM.docx]

|  | **Approval Number** |  |
| --- | --- | --- |
| Hamburg, Asklepios Klinik St. Georg | 2021-100615-BO-ff |  |
|  |  |  |
|  |  |  |
|  |  |  |
|  |  |  |
|  |  |  |
| Sheffield Teaching Hospital, NHS | IRAS #301571 |  |
| Vienna, University Hospital | 2036/2021 |  |
|  |  |  |
| Lund, Skane University Hospital | Dnr 2022-02888-01 |  |
| Harefield Hospital, London | IRAS #301571 |  |
| Heidelberg, University Hospital | S-160/2022 |  |
| Ulm, University Hospital | 483/21 |  |
|  |  |  |
|  |  |  |
| Essen, University Hospital | 21-10373-BO |  |
|  |  |  |
|  |  |  |
| München, German Heart Center | 124/22 S |  |
|  |  |  |
|  |  |  |
| Erlangen, University Hospital | 22-74-Bn |  |
|  |  |  |
| Hannover, MHH | 10353_BO_K_2022 |  |

**Definition of Adverse Device Effects (ADE)**

According to ISO 14155:2020 chapter 3.1, an ADE is defined as an adverse event related to the use of an investigational medical device.

This definition includes AE resulting from insufficient or inadequate IFU, deployment, implantation, installation, or operation, or any malfunction of the MD. This definition also includes any event resulting from use error or from intentional misuse of the MD.

All Investigators were asked to assess the relationship of the MD or procedure to the AE. The following categories for classification of relationship are available (acc. to MEDDEV 2.7/3 revision 3, May 2015, ref.16):

A causal relationship towards the MD or study procedure should be rated as follows:

• Not related: The relationship to the device or procedures can be excluded.

• Unlikely: The relationship with the use of the device seems not relevant and / or the event can be

reasonably explained by another cause, but additional information may be obtained.

• Possible: The relationship with the use of the device is weak but cannot be ruled out completely.

Alternative causes are also possible.

• Probable: The relationship with the use of the device seems relevant and / or the event cannot

reasonably explained by another cause.

• Causal relationship: The serious event is associated with the device or with procedures beyond

reasonable doubt.

**Definition of Serious Adverse Device Effects (SADE)**

A SADE is defined as an ADE that has resulted in any of the consequences characteristic for a SAE (as per ISO 14155:2020, chapter 3.44).

Definition of Unanticipated Serious Adverse Device Effect (USADE)

An USADE is defined as a SADE which by its nature, incidence, severity or outcome has not been identified in the current risk assessment (see ISO 14155:2020, chapter 3.51). SADE and USADE will immediately be analyzed by the sponsor in regard to possible root-cause and possibility of reoccurrence. In addition, all statutory reporting duties will be fulfilled. In case of USADE, the

investigator has the same notification duties as for SADE.

• Date

• Event category

• UADE (if applicable)
